# Supplementary material for: Interactions between the 2C protein of FMDV and components of the viral replication machinery are mediated by ER-derived membranes
Source: bioRxiv. 2025 Sep 17:2025.09.17.676763. Preprint. [Version 1] doi: 10.1101/2025.09.17.676763 (PMC12458404; doi:10.1101/2025.09.17.676763)
Supplement: Supplement 1 [file media-1.pdf]

Supplementary information

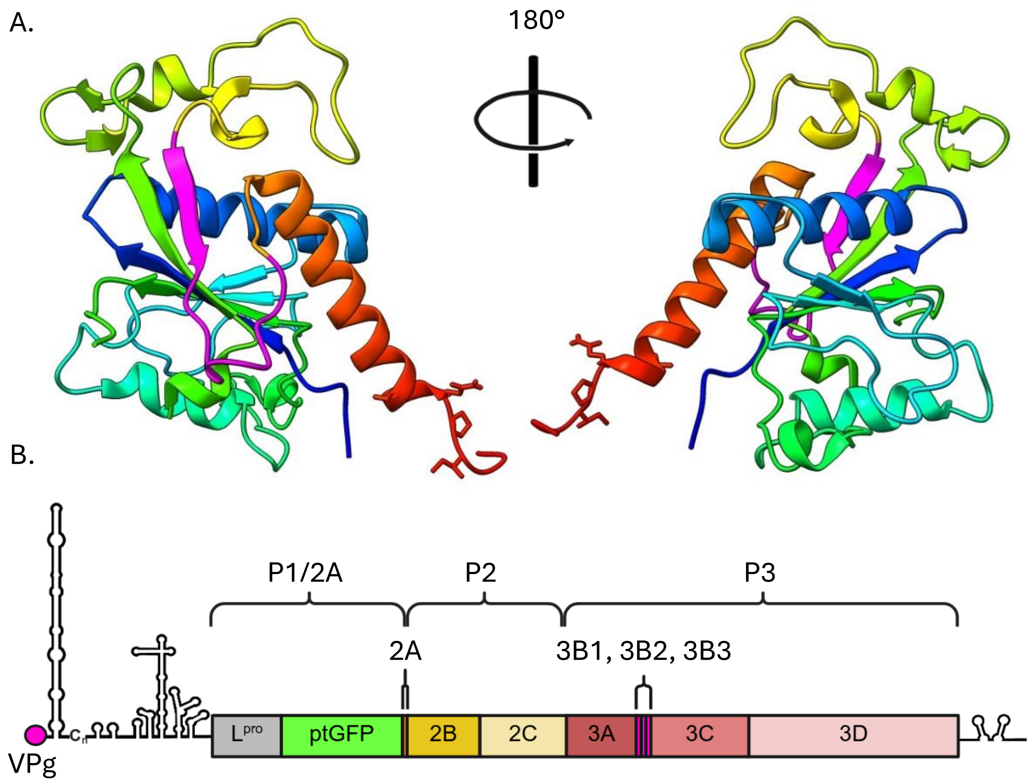

Figure S1. (A) Peptide residues (pink) used to generate novel anti-2C antibody mapped onto the crystal structure of FMDV protein residues 97-318 aa (PDB 7E6V), coloured N- to C- terminal rainbow gradient (Zhang *et al.*, 2022).

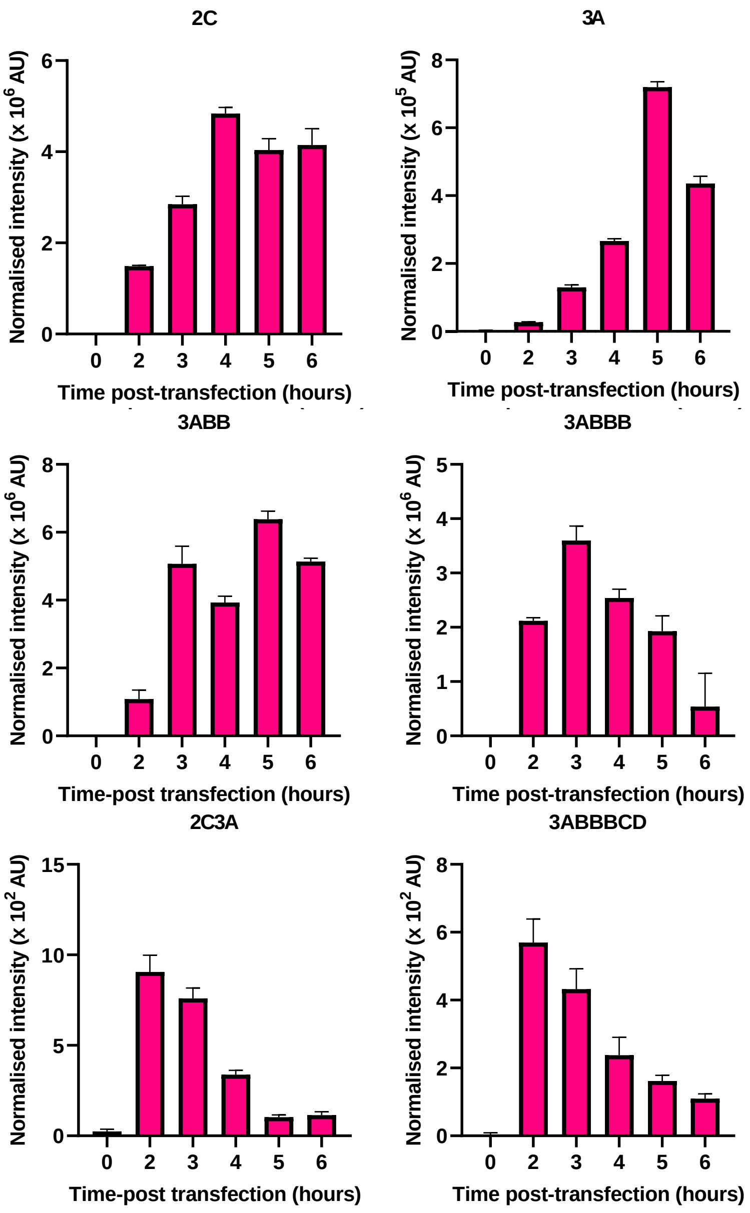

Figure S2. Densitometry of FMDV protein expression over time.

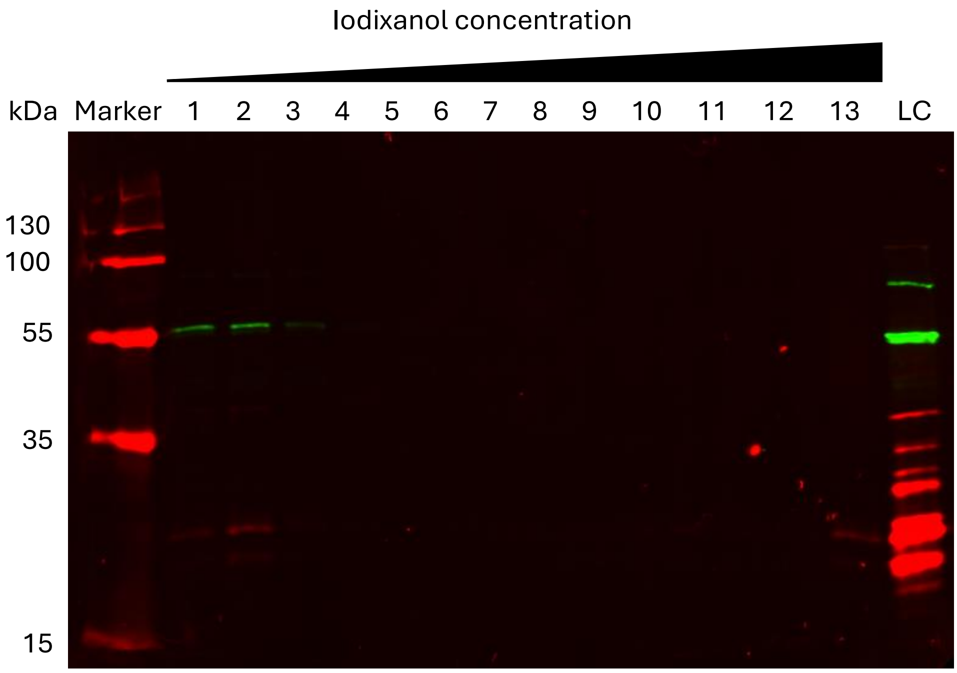

Figure S3. Primary gradient purification of GNN transfected lysate. Probed with anti-3D and anti-3A. LC = Loading control of wild-type transfected lysate.

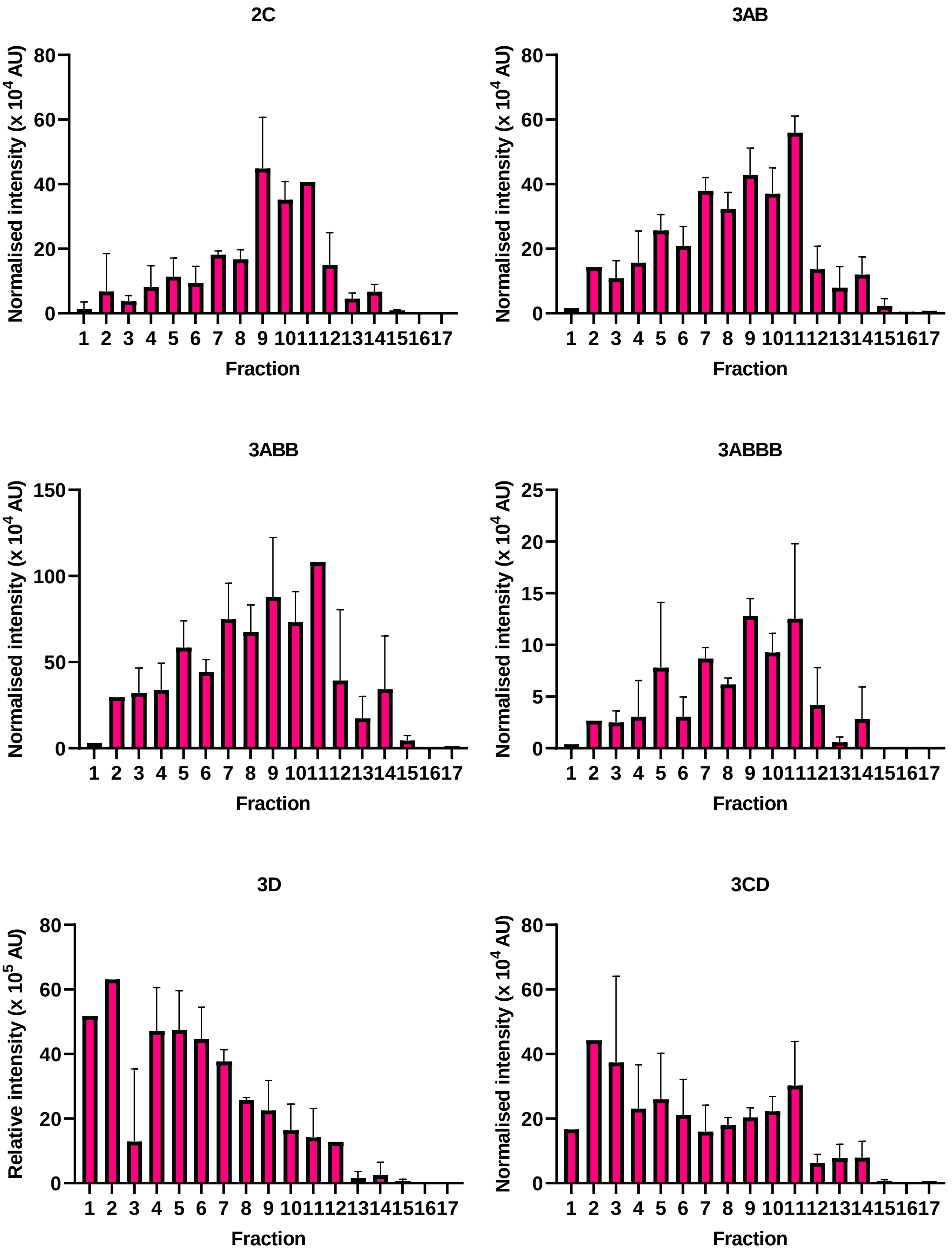

Figure S4. Densitometry of FMDV protein signal across a primary gradient purification. Normalised to loading control of wild-type transfected lysate.

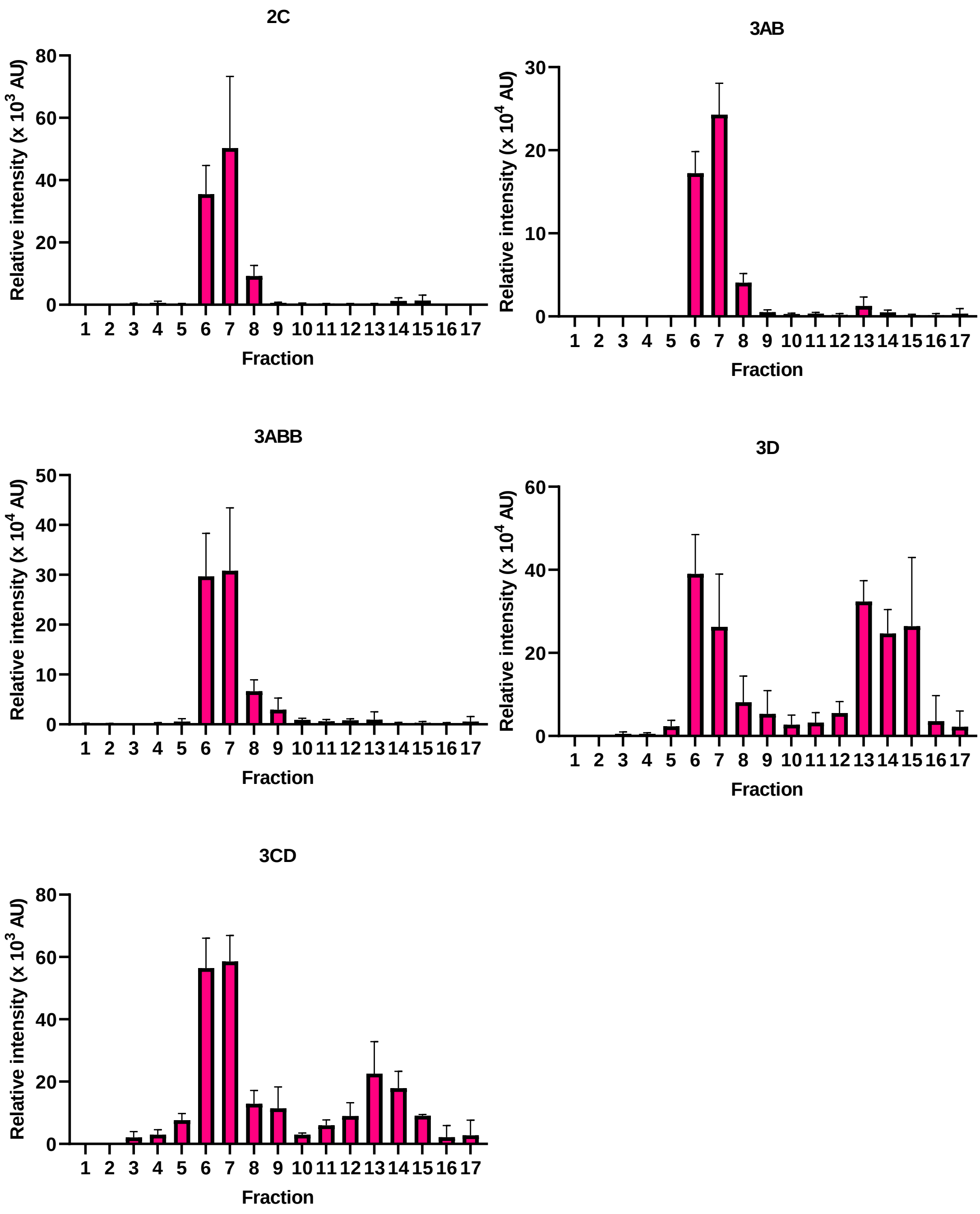

Figure S5. Densitometry of FMDV protein signal across secondary gradient purification. Normalised to loading control of wild-type transfected lysate.
